# Validation of the Korean Version of the Brief Pain Catastrophizing Scale in Patients with Chronic Pain

**DOI:** 10.1192/j.eurpsy.2025.547

**Published:** 2025-08-26

**Authors:** H. Yang, J. M. Park, S. B. Cho, S. Y. Lee, C. Noh, C. Roh, S. Cho

**Affiliations:** 1 Department of Psychology, Chungnam National University, Deajeon; 2 Department of Psychology, Pusan National University, Pusan; 3 Department of Anesthesia & Pain Medicine, Chungnam National University School of Medicine, Deajeon, Korea, Republic Of

## Abstract

**Introduction:**

The Pain Catastrophizing Scale (PCS) is a widely used self-report tool for evaluating pain-related catastrophizing. In response to the need for more efficient diagnostic tools in clinical environments, the PCS has been shortened from 13 to 4 items in developing the brief version.

**Objectives:**

The objectives of this study were: (1) to examine the factor structure of a Korean-language version of the brief K-PCS and (2) to assess the reliability and validity of the brief K-PCS.

**Methods:**

A total of 131 patients seeking treatment at a tertiary pain center in Daejeon, Korea, participated. Confirmatory factor analysis (CFA) with maximum-likelihood estimation was performed to evaluate the adequacy of the one-factor model. Cronbach’s alpha coefficients and Pearson correlations were calculated to investigate internal consistency and 2-week test-retest stability of the brief K-PCS, respectively. For concurrent validity, Pearson correlations were also calculated to examine the relationships between the brief K-PCS and various outcome measures.

**Results:**

The confirmatory factor analysis confirmed the adequacy of the brief K-PCS’s unifactor structure, indicated by excellent fit indices (CFI = .999, TLI = .996, SRMR = .039). The brief K-PCS exhibited high internal consistency (Cronbach’s α = .83). Test-retest correlations over a 2-week interval was .744 (p < .001), indicating high stability. For concurrent validity, the brief K-PCS showed significant positive correlations with measures of depression, fearful thinking, physical response, avoidance, and pain-related anxiety (p < .001), and significant negative correlations with quality of life measures, including physical, psychological, social relationships, environmental, and general quality of life (p < .001).

**Conclusions:**

The brief K-PCS is a reliable and valid tool for assessing pain catastrophizing in a Korean patient sample with chronic pain.

**Disclosure of Interest:**

H. Yang Grant / Research support from: National Research Foundation of Korea Grant funded by the Korean Government(NRF-2022S1A5A2A03050752), J. M. Park Grant / Research support from: National Research Foundation of Korea Grant funded by the Korean Government (NRF-2022S1A5A2A03050752), S. B. Cho Grant / Research support from: National Research Foundation of Korea Grant funded by the Korean Government (NRF-2022S1A5A2A03050752), S. Y. Lee Grant / Research support from: National Research Foundation of Korea Grant funded by the Korean Government (NRF-2022S1A5A2A03050752), C. Noh Grant / Research support from: National Research Foundation of Korea Grant funded by the Korean Government (NRF-2022S1A5A2A03050752), C. Roh Grant / Research support from: National Research Foundation of Korea Grant funded by the Korean Government (NRF-2022S1A5A2A03050752), S. Cho Grant / Research support from: National Research Foundation of Korea Grant funded by the Korean Government (NRF-2022S1A5A2A03050752)

